# A New Cause of Death of Cattle

**Published:** 1895-08

**Authors:** N. S. Mayo


### A NEW CAUSE OF DEATH TO CATTLE.[1]

BY N. S. MAYO, D.V.S.

I HOPE you will not think I am presuming too much or am egotistical in selecting such a title as this for my paper, but it is the only name which seems to apply, and by the word *new* I only mean a cause of death which is new to me. I have searched diligently through all the literature at my command and have failed to find such a case reported. With this explanation I hope you will pardon my seeming egotism which at first might impress you.

There are least three distinct causes of death among cattle when pastured upon cornstalks or sorgum. There is the widely-

[1] Read before the June meeting of the Missouri Valley Veterinary Medical Association.

recognized "cornstalk disease," with which you are all more or less familiar. Then there is the sorgum, especially the second growth, which under certain circumstances will certainly kill cattle, and very quickly; and, lastly, there is a disease of which I shall speak.

The history of the cases is as follows: William Errickson, a Swede, living near Olsburg, Kansas, on the 17th of last August, about 5 o'clock in the afternoon, brought up to his barnyard twelve head of cattle, cows and young stock. As he was going away for the evening, and had taken them up earlier than usual, he fed them some cornstalks, estimated at four large armfuls, putting the stalks in piles about the yard. The pastures being very dry, the cattle ate the stalks freely. The stalks had been cut some time previously. Mr. Errickson returned home about 1 o'clock A.M., and in passing through the yard noticed something peculiar about his cattle, and on investigation found that seven out of the twelve head were dead—lying in easy positions in various parts of the yard—having died with little or no struggling, as far as he could determine. Five head were milch cows, one yearling steer, and one yearling heifer. Some of them were bloated the next morning, caused probably by the gases of decomposition.

One animal (the heifer) was brought to Manhattan just as I was leaving for the western part of the State, but as the train was a few minutes late I had an opportunity to open the ventral cavity only. The abdominal cavity contained a large amount of serum. The rumen was highly congested over quite large areas, almost, if not quite, inflamed. The rumen contained a small amount of corn-fodder. The reticulum was badly congested, as was also the abomasum. Liver congested, lungs congested (partially hypostatic). The œsophagus contained a small quantity of egesta, evidently vomited. Other organs normal, as far as a hasty examination showed.

Mr. Errickson the next morning after losing the cattle called his family physician (a graduate), who informed me that all the cattle had evidently tried to vomit some. He said the lungs were congested, but the *trocar* was all right (meaning the trachea). Mr. Errickson suspected that his cattle had been poisoned, and his physician confirmed his suspicions by saying that it was undoubtedly strychnine poisoning: a most absurd conclusion from the natural positions the animals assumed dying.

I myself suspected poisoning, I could lay it to nothing else; so portions of the various organs and contents of the rumen were taken and analyzed for all the common poisons, but without result. Professor Failyer, chemist at this college, had investigated the cause of death of a number of cattle several years ago at Grainfield, and said he had found a large amount of nitrate of potash in the stalks, and suspected this to be the cause. I immediately wrote to Mr. Errickson to find out where the stalks were grown and for samples of stalks. He said the stalks were grown in an old hog-lot where hogs had been kept several years. The samples of stalks sent contained 25 per cent. of their weight pure nitrate of potash. The potash was so abundant that it had crystallized on the outside of the stalks like a white mould, and the stalks themselves when lighted with a match burned like the fuse of a fire-cracker. To test the amount necessary to cause death, a heifer, weighing about five hundred pounds, was given 300 grains (9¾ ounces) of nitrate of potash as a drench. In ten minutes she commenced to urinate, and in fifteen minutes there was a violent trembling of the voluntary muscles, the animal falling down. She continued to urinate about every ten minutes for an hour. Her temperature fell ½° F. in twenty minutes, but after an hour commenced to rise again. It was thought from her general appearance (she fell down in about twenty minutes and was unable to rise) that she would die immediately, but after two hours she commenced to improve, and it was supposed she would recover, but she died during the night. An autopsy revealed the same extensive congestion and inflammation of the rumen, and especially of the abomasum. I also tested nitrate of potash on rabbits and guinea-pigs, and can kill them in twenty minutes. The potash kills apparently by a paralysis of the respiratory and circulatory centres. I am certain that twelve ounces of nitrate of potash will kill a heifer of five-hundred-pound weight in thirty minutes. So these cattle in eating four pounds of stalks would get one pound of nitrate of potash. So it seems conclusive that the cattle died from the effects of the nitrate of potash in the cornstalks.

Some interesting and important points are as follows: First, The rapidity of the action of nitrate of potash in large doses. Effects shown in ten minutes by the watch—no guessing—and the comparatively small amount necessary to kill an animal.

Nitrate of potash will only be found in cornstalks grown on *very rich soil*, such as feed-lots and yards which contain a large amount of nitrites in the soil. I am of the opinion that potash will be found in large quantities in stalks only in very dry seasons. Still, this is only a theory, and may not prove true. I reason that in a wet season the potash, being readily soluble, would be washed away by the rains.

I trust that this may help others to account for some of the many mysterious causes of death which often come to the practising veterinarian, and hope I have not wearied you with this long and tedious dissertation.

